# Integrated Metabolomic and Transcriptomic Analysis of the Flavonoid Accumulation in the Leaves of *Cyclocarya paliurus* at Different Altitudes

**DOI:** 10.3389/fpls.2021.794137

**Published:** 2022-02-08

**Authors:** Zhaokui Du, Weida Lin, Binbin Yu, Jinxing Zhu, Junmin Li

**Affiliations:** ^1^Zhejiang Provincial Key Laboratory of Plant Evolutionary Ecology and Conservation, Taizhou University, Taizhou, China; ^2^Taizhou Vocational College of Science and Technology, Taizhou, China; ^3^Suichang County Bureau of Agriculture and Rural Affairs, Lishui, China

**Keywords:** flaovnoid, metabolome, transcriptome, altitude, *Cyclocarya paliurus*

## Abstract

*Cyclocarya paliurus* is a medicinal plant containing flavonoids, triterpenoids, polyphenolics, polysaccharides, and other compounds with diverse biological functions. *C. paliurus* is distributed across altitudes ranging from 400 to 1,000 m. However, little is known about the effect of altitude on metabolite accumulation in *C. paliurus.* Also, the biosynthetic pathway involved in flavonoid accumulation in *C. paliurus* has not been fully elucidated. In this study, mature leaves of *C. paliurus* growing at low altitude (280 m) and high altitude (920 m) were sampled and subjected to metabolomic and transcriptomic analyses. The flavonoid content and composition were higher in the leaves of *C. paliurus* collected at high altitude than in those collected at low altitude. Most of the differentially accumulated metabolites (DAMs) were enriched in “flavone and flavonol biosynthesis.” The significant differentially expressed genes (DEGs) between low and high altitudes were mainly enriched in “biological process.” The most heavily enriched KEGG pathway was related to the subcategory “Oxidative phosphorylation,” indicating that complicated biological processes are involved in the response of *C. paliurus* to harsh environmental factors. High UV-light might be the main influencing factor among the harsh environmental factors found in high altitudes. Integrated analysis of metabolomic and transcriptomic data showed that 31 flavonoids were significantly correlated with 227 DEGs, resulting in 412 related pairs (283 positive and 129 negative) between the DEGs and flavonoids. The possible mechanisms underlying the differentially accumulation of flavonoids at different altitude might be due to variations in transport and relocation of flavonoids in *C. paliurus* leaves, but not different flavonoid biosynthesis pathways. The up-regulation of genes related to energy and protein synthesis might contribute to flavonoid accumulation at high altitudes. This study broadens our understanding of the effect of altitude on metabolite accumulation and biosynthesis in *C. paliurus.*

## Introduction

Several environmental factors are altered at different altitudes, including night/day temperature, UV radiation, light intensity, among others (Voronkov et al., 2019). The harsh environmental conditions at high altitudes can induce complicated responses in plants (Ma et al., 2015; Zhang et al., 2019; Kumari et al., 2020; Yu et al., 2021), insects (Liu et al., 2020), birds (Hao et al., 2019), and mammals (Tang et al., 2017). High-throughput molecular biological techniques (such as multi-omics approaches) and morphological statistics have been widely applied to explore plant responses to high-altitude environments. For example, a combination of morphological, biochemical, and proteomics analysis revealed that primary metabolites, antioxidant proteins, and proteins associated with the epigenetic regulation of DNA stability and post-translational protein degradation are involved in the adaptation of *Potentialla saundersiana* to high altitude environments (Ma et al., 2015). The study uncovered the mechanisms underlying plant response to high altitude and was instrumental in understanding the adaptative strategies of plants to complicated environmental factors.

Medicinal plants are rich in bioactive phytochemicals that are beneficial to human health (Kumari et al., 2020). However, medicinal plants encounter various environmental stresses during their growth and development in the field, leading to metabolic, morphological, and physiological changes. Therefore, studying the metabolomics of plants growing in natural habitats under different environmental variables is crucial for a broader understanding of metabolite biosynthesis. Kumari et al. (2020) analyzed metabolite accumulation in *Picrorhiza kurroa* plants (at the flowering stage) collected from three altitudes, viz. 3,400, 3,800, 4,100 m in the Himalayan region using LC-MS based non-targeted metabolite profiling and targeted analysis of sugars, amino acids, picrosides, and their corresponding phenolic acids, and found that metabolite accumulation varied with altitude. High altitude has been shown to induce anthocyanidin accumulation and changes in 50 metabolites from seven groups, including carbohydrates, amino acids, organic acids, lipid components, polyamine, secondary metabolism, and others in *Herpetospermum pedunculosum* (Zhao et al., 2019). It also increases the activities of ascorbate peroxidase, glutathione reductase, dehydroascorbate reductase, monodehydroascorbate reductase, and superoxide dismutase in *H. pedunculosum* (Zhao et al., 2019). However, information about how metabolic constituents of medical plants respond to changes in altitude is scanty. Thus, there is a need to conduct multi-omic analysis to uncover the underlying molecular pathway of metabolite biosynthesis in medicinal plants found at high altitudes.

*C. paliurus* has various medicinal properties, including hepatoprotective, anti-inflammatory, immune-stimulatory, and free radical scavenging activity (Zhou et al., 2019b). Extracts from its leaves contain flavonoids, triterpenoids, polyphenolics, polysaccharides, and other compounds that exhibit diverse biological functions, including antioxidant, antimicrobial, and antidiabetic activities (Liu et al., 2018; Yang et al., 2018; Zhou et al., 2019a). *C. paliurus* is distributed across altitudes ranging from 400 to 1,000 m (Mo et al., 2014). The metabolites in the leaves of *C. paliurus* vary with altitude and plant location. Wu (2019) found that the contents of polysaccharides, flavonoids, and terpenoids in *C. paliurus* leaves from Anhui Province were highest at altitudes of 600–800 m. Wang et al. (2020) compared the flavonoids and polyphenol contents in *C. paliurus* leaves grown at 400 m and 1,300 m in Suicang County, Zhejiang Province, and found that those at 1,300 m were significantly higher than at 400 m. Mi et al. (2009) found that the flavonoids content in *C. paliurus* leaves in Xiushui County, Jiangxi Province, showed sawtooth form with the increase in altitude, and the highest flavonoid content was found in leaves collected at 915 m. In our previous studies, we combined metabolomic and transcriptomic data to explore the biosynthetic pathway of phenolic acid (Lin et al., 2020), polysaccharides (Lin et al., 2021), and flavonoids (Sheng et al., 2021) in the leaves of *C. paliurus*. However, little is still known about the effect of altitude on the metabolite content of *C. paliurus* leaves. Besides, the possible biosynthetic pathway of metabolite accumulation in *C. paliurus* at different altitudes has not been fully elucidated. In this study, metabolic and transcriptomic analyses were conducted on mature leaves collected from *C. paliurus* growing at low and high altitudes (280 m vs. 920 m) in Longquan City, Zhejiang Province to determine (1) the effect of altitude on flavonoid content of the leaves of *C. paliurus*; (2) the effect of altitude on metabolite accumulation in the leaves of *C. paliurus*; (3) the effect of altitude on gene expression in the leaves of *C. paliurus*; (4) and the possible mechanisms of flavonoid biosynthesis and accumulation in the leaves of *C. paliurus* at different altitudes. Our results provide new insights into the molecular mechanisms of phytochemical accumulation in *C. paliurus* at different altitudes.

## Materials and Methods

### Plant Materials

In the Spring of 2015, 1-year-old seedlings of *C. paliurus* were purchased from Yongfeng Town, Shuangfeng County, Hunan Province, China. Subsequently, the seedlings were transplanted on a field basement of Longquan at low altitude (E119°1′10″, N28°4′78′, 280 m) in Xiajin Village and high altitude (E118°48′28″, N28°5′57″, 920 m) in Zhuzhang Village, Longquan City, Zhejiang Province, China. Longquan city is located in the middle subtropical monsoon climate zone and the detailed climate data were shown in Supplementary Table 1. It is characterized by four distinct seasons, abundant rainfall, no severe cold in winter, no intense heat in summer, early spring and long summer, warm and humid. On May 1, 2018, mature leaves with the largest leaf area at F4 stage (Lin et al., 2020, 2021; Sheng et al., 2021) were collected from *C. paliurus* plants growing at the two locations. The leaves collected from high altitude were significantly larger and longer than those collected from low altitude (Supplementary Figure 1 and Supplementary Table 2). The leaves from various plants in different regions were separately sampled simultaneously, as described by Lin et al. (2020). The leaves from three randomly selected plants were considered as three biological replicates. The leaf samples were divided into two sub-samples. One sub-sample was immediately frozen in liquid nitrogen and stored at –80°C for metabolomic and transcriptomic analyses. The other sub-sample was dried at 70°C to a constant weight, then ground to a fine powder and sieved through a 40-mesh to determine flavonoids contents.

### Measurement of Flavonoid Contents

To extract flavonoid, we dissolved 2 g of the ground *C. paliurus* leaves in 25 mL of 70% ethanol and incubated the solution at 70°C for 60 min. Next, the solution was centrifuged at 4,000 rpm for 15 min. Subsequently, the supernatant (0.5 mL) was transferred to a test tube, and 0.15 mL of 5% NaNO_2_ solution was added, and the mixture was incubated at room temperature for 5 min. Then, 0.15 mL of 10% AlCl_3_• 6H_2_O solution was added to the mixture and incubated at room temperature for 5 min. After that, 1 mL of 1 mol/L NaOH solution was added, and the mixture was further incubated at room temperature for 15 min. Finally, TU-1901 UV -Vis Spectrometer (Beijing Persee General Instrument Co., Ltd.) was used to measure the absorbance of the solution at 415 nm. The standard calibration curve of rutin was obtained using the following linear regression equation: *y* = 3.145 × –0.0072 (*R*^2^ = 0.9993).

Statistical analyses were performed using SPSS Statistics 20 (IBM Corp., Armonk, NY, United States). Each value represents the mean ± SD (standard deviation) of three independent biological replicates. Differences in flavonoid content between samples collected from different altitudes were evaluated using a one-way analysis of variance (ANOVA).

### Metabolomic Analysis

The freeze-dried leaves of *C. paliurus* were ground in a PM400 high-energy ball mill (Retsch Technology GmbH, Haan, Germany), then used for metabolomic analysis. Metabolites were extracted from the powder (100 mg) according to the methods described by Lin et al. (2020). Ultra-Performance Liquid Chromatography (UPLC; Shim-pack UFLC SHIMADZU CBM30A) and Tandem mass spectrometry (MS/MS; Applied Biosystems 6500 QTRAP) were conducted by Metware Biotechnology Co., Ltd. (Wuhan, China). Multiple reaction monitoring (MRM) was performed as previously described (Lin et al., 2020). Metabolites with variable importance in projection (VIP) ≥ 1 and fold changes ≥ 2 or ≤ 0.5 were defined as significantly different accumulated metabolites (DAMs) in content. The MRM of each species was repeated three times. Principal component analysis (PCA) and heatmap cluster analysis were performed to assess the difference in DAMs between low and high altitudes (Sheng et al., 2021).

The identified metabolites were annotated using KEGG (Kyoto Encyclopedia of Genes and Genomes) compound database.^1^ Annotated metabolites were then mapped to the KEGG pathway database.^2^ Significantly enriched pathways were identified using a hypergeometric test’s *p*-value for a given list of metabolites.

### Transcriptomic Analyses

Total RNA was extracted from freeze-dried leaves of *C. paliurus* using Trizol reagent, according to the manufacturer’s instructions. Subsequently, the concentration, fragment size, and integrity of the extracted RNA were measured using an Agilent 2100 bioanalyzer (Agilent Technologies Inc., California, United States). The cDNA libraries of leaves at low and high altitudes were constructed and sequenced by Tianjin Novogene Co., Ltd., using an Illumina HiSeq™ 3000 platform (100 bp paired-end sequencing). Three replicates were used for each altitude. All raw data have been deposited at the National Center for Biotechnology Information (NCBI) database under accession number PRJNA769609.

The raw data were further processed as described previously (Lin et al., 2020). The obtained clean data was used to calculate the Q20, Q30, and GC content. BLAST program was used to annotate the functions of unigenes against protein databases, including NCBI Nr (NCBI non-redundant protein sequences), Swiss-Prot protein, TrEMBL, ConserGene Ontology (GO), CDD (Conserved Domain Database), Pfam, and KOG (eukaryotic Orthologous Groups) database (*e*-value < 1 × 10^–5^). GO (Gene Ontology Database) function annotation information was obtained according to the Uniprot annotation results based on the annotation results of Swiss- + Prot and TrEMBL database. KEGG annotation was conducted by KAAS (KEGG automatic annotation server) (version 2.1). All the assembled unigenes were classified into KEGG pathways using BLASTX against the KEGG database (Zhang et al., 2018). Differentially expressed genes (DEGs) in the leaves of *C. paliurus* grown at low and high altitudes were filtered using a threshold of absolute fold change of log_2_ ≥ 1 with *q*-value ≤ 0.05.

### Real-Time Quantitative PCR Validation of RNA-Seq Data

The expression levels of eight selected genes were determined using real-time quantitative PCR (RT-qPCR) to verify the expression patterns of the mRNAs identified by RNA-seq. RT-qPCR was performed on CFX Connect (Bio-Rad Laboratories Inc. Hercules, CA, United States) using HiScript II reverse transcriptase (Vazyme Biotech Co. Ltd., Nanjing, China), according to the manufacturer’s protocol. The primers used for RT-qPCR are shown in Supplementary Table 3. Sample cycle threshold (Ct) values were determined and normalized against the constitutively expressed gene (β*-actin-1*, internal control) (Zhao et al., 2018). The 2^–ΔΔCT^ method was used to calculate the relative gene expression level. Three biological replicates and three technical replicates were used for all RT-qPCR reactions.

### Integrative Analysis of Metabolomic and Transcriptomic Data

Pearson correlation coefficient analysis was performed based on the DAMs and DEGs to integrate the metabolome and transcriptome data. The screening criteria were a *P*-value < 0.01 and a *r*-value > 0.9. The mean of all biological replicates for each color morph in the metabolome data and the mean value of expression of each transcript in the transcriptome data were calculated. Subsequently, the relationships were visualized using the Cytoscape software (version 2.8.2).

## Results

### Effect of Altitude on Flavonoid Accumulation in *Cyclocarya paliurus*

**FIGURE 1:**
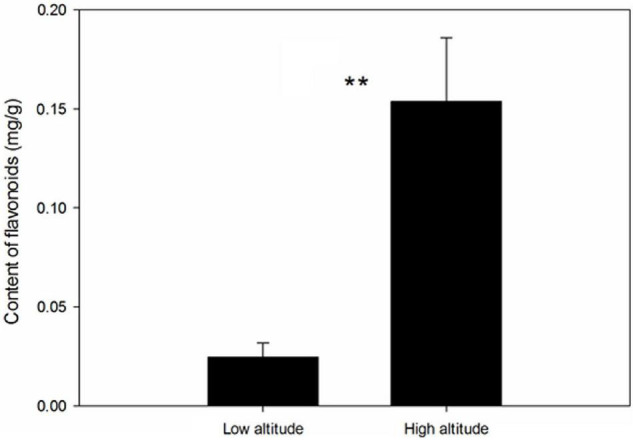
Effect of altitude on flavonoid content in the leaves of *Cyclocarya paliurus*. ** Indicates significant difference at *P* < 0.01.

Altitude had a significant effect on the metabolite content of the leaves, with plants collected at high altitude having higher levels of flavonoids than those collected at low altitude (Figure 1, *F* = 46.26, *p* = 0.002).

### Comparison of Metabolomic Data

**FIGURE 2:**
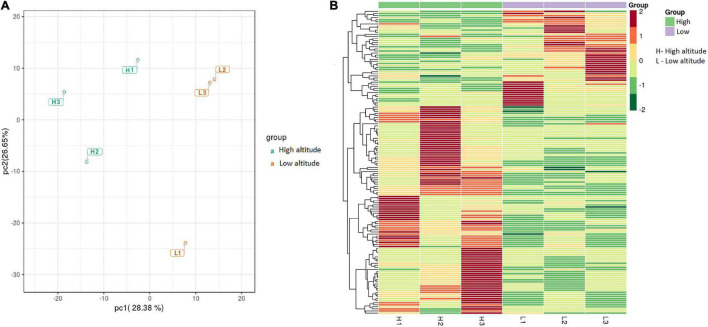
Principal component analysis (PCA) **(A)** and heatmap **(B)** of differentially accumulated metabolites (DAMs) in the leaves of *Cyclocarya paliurus* between low altitude (L1∼L3) and high altitude (H1∼H3). The log_2_ transformed values of DAMs are indicated from green to red (low to high).

Significant differences in the metabolite content of the leaves of *C. paliurus* were found between low and high altitudes based on PCA analysis (Figure 2A) and heatmap (Figure 2B). Among the 685 metabolites detected by LC-MS/MS, 146 significant DAMs were found between low and high altitudes and were classified into 16 categories (Table 1). The top five compounds were ranked in the orders: flaovnoids including anthocyanins (66 compounds), lipids (19 compounds), amino acids and its derivatives (19 compounds), organic acids (9 compounds), and nucleotide derivatives (8 compounds). Among them, 100 DAMs were higher in the leaves of *C. paliurus* grown at high altitude than at low altitude, while 46 exhibited the opposite trend (Table 1 and Supplementary Table 4), indicating that *C. paliurus* grown at high altitude have more and diverse metabolites than those at low altitude.

**TABLE 1:** Metabolite accumulation in the leaves of *Cyclocarya paliurus* at low and high altitudes.

Order	Group	Number of compounds	HA > LA	LA > HA
1	Flavonoids (including anthocyanins)	66	47	19
2	Lipids	19	16	3
3	Amino acids and its derivatives	19	8	11
4	Organic acids	9	5	4
5	Nucleotide derivatives	8	4	4
6	Hydroxycinnamoyl derivatives	6	3	3
7	Phenolamides	4	4	0
8	Coumarins	3	2	1
9	Tryptamine derivatives	3	3	0
10	Indole derivatives	2	2	0
11	Alcohols and polyols	2	2	0
12	Vitamins	1	1	0
13	Alkaloids	1	1	0
14	Cholines	1	1	0
15	Quinate and its derivatives	1	0	1
16	Others	1	1	0
	Total	146	100	46

*HA indicates high altitude, while LA indicates low altitude.*

**FIGURE 3:**
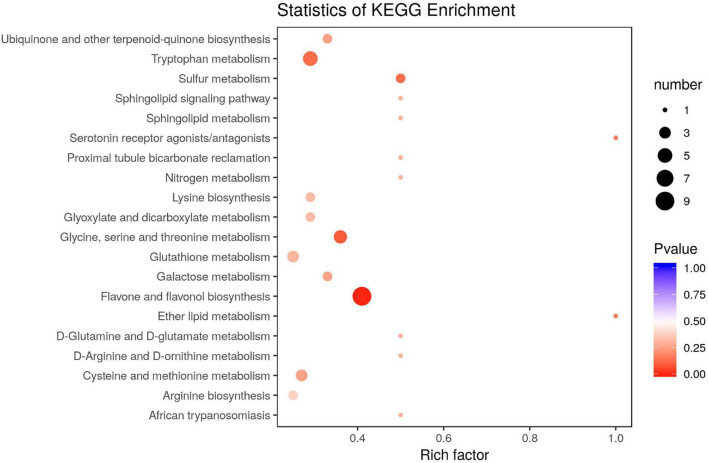
KEGG pathway enrichment of differentially accumulated metabolites (DAMs) in the leaves of *Cyclocarya paliurus* between low and high altitudes.

The KEGG pathway enrichment of the DAMs revealed that most DAMs were mapped onto “flavone and flavonol biosynthesis,” “tryptophan metabolism,” “glycine, serine, and threonine metabolism,” and “sulfur metabolism” pathways, among others (Figure 3).

Sixty-six flavonoids detected in the leaves of *C. paliurus* growing at low and high altitudes was divided into more than 17 groups, including chrysin, apigenin, chrysoeriol, and their derivatives (Supplementary Table 5). Among them, gallocatechin was the most abundant flavonoid in the leaves of *C. paliurus*, while luteolin 7-O-glucoside took the second place (Supplementary Table 5). In addition, 47 metabolites were higher at high altitude than at low altitude, and 19 exhibited the opposite trend when the threshold for significant differences was set at VIP ≥ 1 and fold changes ≥ 2 or ≤ 0.5 (Table 1 and Supplementary Table 5). For example, chrysoeriol 7-O-rutinoside, naringin, tricin 7-O-β-guaiacylglycerol, tricin O-malonyl rhamnoside were mainly found in the leaves collected at high altitude, and in contrast, eriodictiol C-hexosyl-O-hexoside was only found in the leaves collected at low altitude; O-methylchrysoeriol 5-O-hexoside, O-methylchrysoeriol 7-O-hexoside, troxerutin, tricin O-sinapolylhexoside, kumatakenin, and velutin were 11.53, 11.19, 15.44, 16.56, 12.13, and 10.94 folds higher at high altitude than at low altitude, respectively (Supplementary Table 5). These results indicated that the accumulation of various flavonoids in the leaves of *C. paliurus* varied at different latitudes.

### Comparison of Transcriptomic Data

A total of 31,222,327,390 sequencing reads, including 249,434,590 raw reads and 225,473,198 clean reads were obtained. The average Q20 and Q30 values were 98.44 and 94.30%, respectively. The average GC content was 54.02% (Supplementary Table 6). Combined with unigenes at different developmental stages, 296,593 unigenes were assembled with 127,566,956 bp in total length and 430.11 bp in average length. The annotation results showed that 58.60% were annotated to the NR database, while 40.10% were annotated to the Swissprot database. Also, 35.32% were annotated to NT database, 19.44% to PFAM database, 49.06% to GO database, 21.59% to KOG database, 30.33% to CDD database, 40.10% to TrEMBL database, and 64.68% sequences were annotated in at least one database.

**FIGURE 4:**
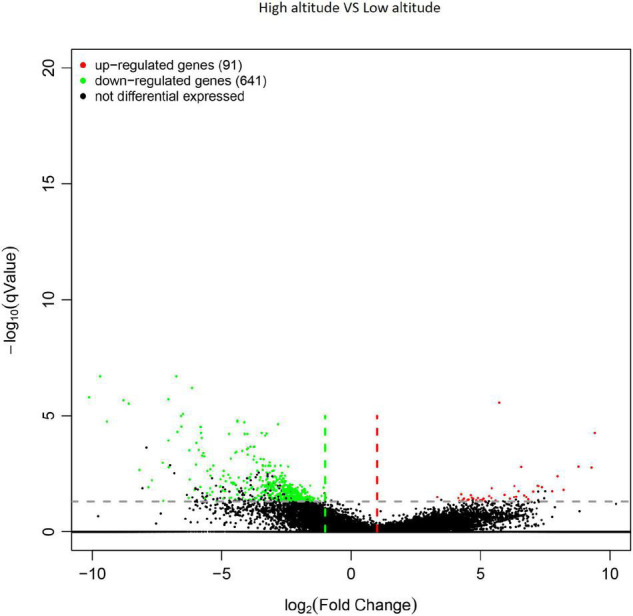
Volcano plot of differentially expressed genes (DEGs) in the leaves of *Cyclocarya paliurus* between low and high altitudes.

In total, 732 genes were obtained from differential expression screening between different altitudes. Among them, 641 genes were down-regulated at high altitude, while the remaining 91 were up-regulated at low altitude (Figure 4).

**FIGURE 5:**
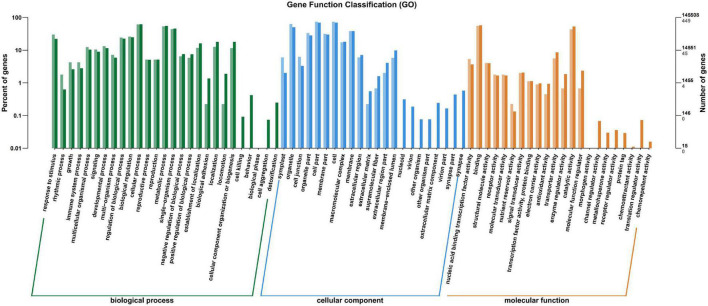
Gene ontology categories of differentially expressed genes (DEGs) in the leaves of *Cyclocarya paliurus* between low and high altitudes.

All the annotated unigenes were divided into 67 functional groups using GO assignments. Among them, 27 groups were involved in biological process category, 22 groups in cellular component category, and 18 groups in molecular function category (Figure 5). Cellular process and metabolic process were dominant in the biological process category. Cell part and cell were dominant in the cellular component category, while binding and catalytic activities were dominant in the molecular function category (Figure 5).

**FIGURE 6:**
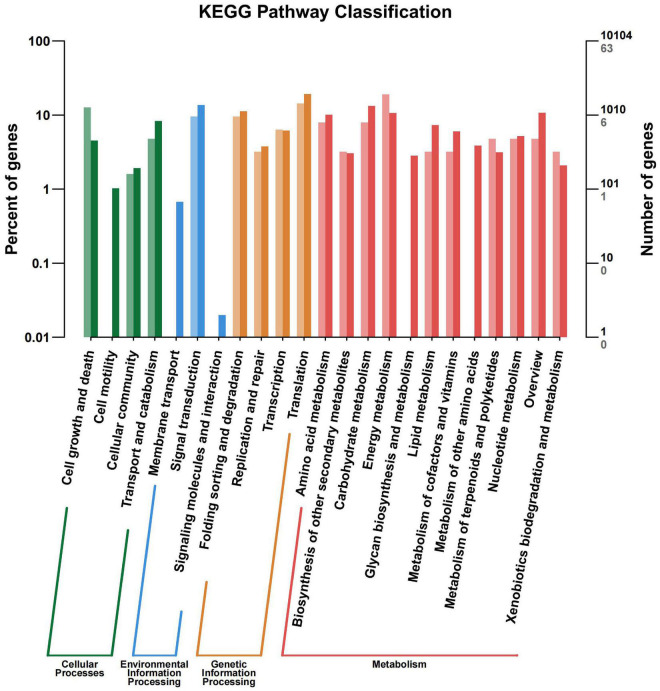
KEGG pathway terms assignment of differentially expressed genes (DEGs) in the leaves of *Cyclocarya paliurus* between low and high altitudes.

**FIGURE 7:**
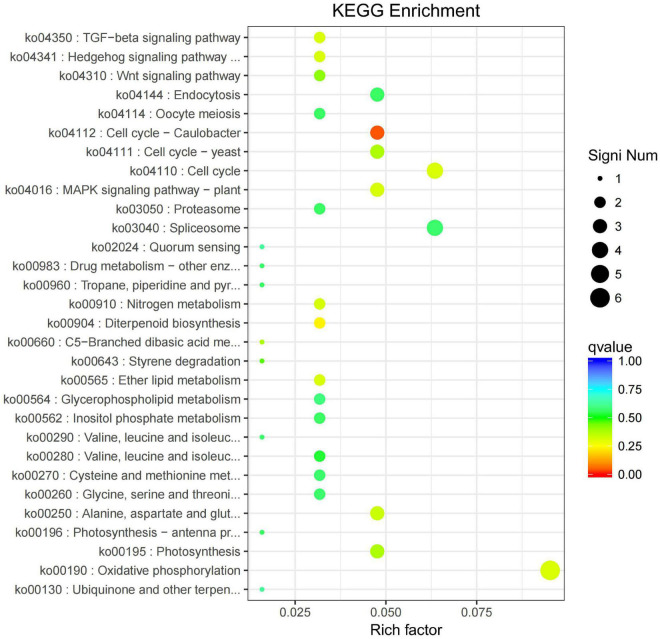
KEGG pathway enrichment of differentially expressed genes (DEGs) in the leaves of *Cyclocarya paliurus* between low and high altitudes.

All the DEGs were assigned into four different KEGG terms in the order from top to down: Metabolisms, Genetic information processing, Cellular processers, and Environmental information processing (Figure 6). The metabolic pathways represented the group, with the most unigenes involved in energy metabolism and carbohydrate metabolism (Figure 6). The top 30 enriched KEGG pathways showed that the most highly enriched KEGG pathway was related to the subcategory “Oxidative phosphorylation” (ko00190), and the next was related to subcategory “cell cycle” (ko04110) and “spliceosome” (ko03040) (Figure 7).

### Flavonoid Accumulation and Biosynthetic Pathway

**FIGURE 8:**
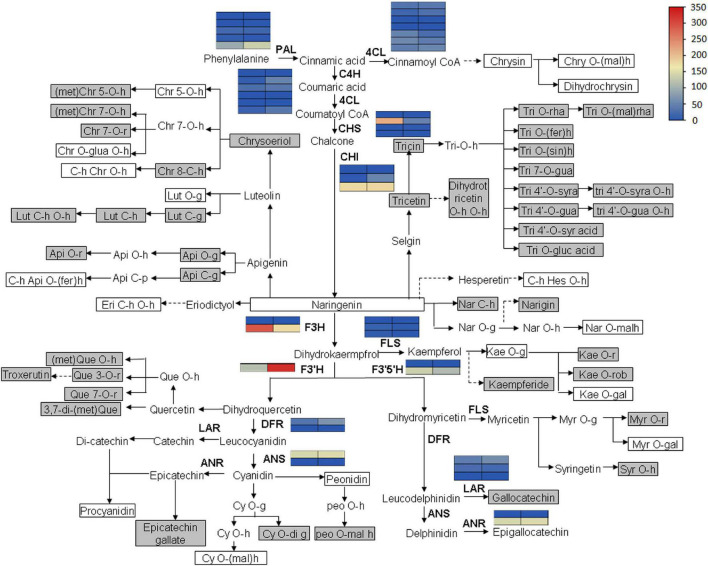
Flavonoid biosynthesis and accumulation pathway in the leaves of *Cyclocarya paliurus* at low and high altitudes. The blue and red colors refer to the expression level of genes from low to high based on fragments per kilobase of exon per million fragments mapped (FPKM) value clustering. The gray box indicates significantly increased metabolites. The white box indicates significantly decreased metabolites. The metabolites without box indicate no significant change. Api, apigenin; Chr, chrysoeriol; Chry, Chrysin; Cy, cyaniding, Dp, delphinidin; Eri, Eriodictyol; fer, feruloyl; gal, galactoside; g, glucoside; gal, galactoside; glua, glucuronic acid; gua, guaiacylglycerol; h, hexoside/hexosyl; Hes, hesperetin; Kae, Kaempferol; Lut, Luteolin; mal, malonyl; met, methyl; Myr, Myricetin; Nar, Naringenin; Peo, Peonidin; Que, Quercetin; r, rutinoside; rha, rhamnoside; rob, robinobioside; rut, rutinoside; sin, sinapolyl; syr, syringic; syra, syringic alcohol; Tri, Tricin; PAL, phenylalanine ammonialyase; 4CL, 4-coumarate coenzyme A ligase; C4H, Cinnamate 4-hydroxylase; CHS, chalcone synthase; CHI, chalcone isomerase; F3H, flavanone 3-hydroxylase; FLS, flavonol synthase; F3′H, flavonoid 3′-hydroxylase; F3′5′H, flavonoid 3′,5′-hydroxylase; DFR, Dihydro flavonol reductase; LAR, Leucoanthocyanidin reductase; ANS, Anthocyanidin synthase; ANR, Anthocyanidin reductase.

**FIGURE 9:**
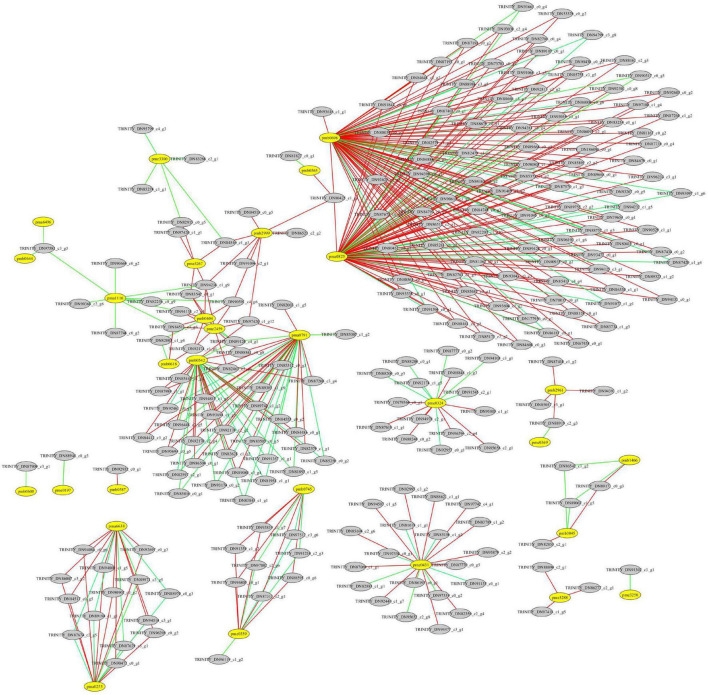
Correlation analysis between differentially expressed genes (DEGs) and differentially accumulated flavonoids in the leaves of *Cyclocarya paliurus* at low and high altitudes. The yellow circle represents flavonoids; the gray circle represents DEGs. The red line indicates a significant positive correlation; the green line indicates a significant negative correlation. pma0253, O-methylchrysoeriol 5-O-hexoside; pma0791, Naringenin O-malonylhexoside; pma0825, chrysin O-malonylhexoside; pma1116, kaempferide; pma6496, luteolin 6-C-glucoside; pma6638, O-methylchrysoeriol 7-O-hexoside; pmb0542, cyaniding 3-O-malonylherxoside; pmb0563, peonidin; pmb0587, chrysoeriol O-glucuronic acid-O-hexoside; pmb0600, chrysoerio 7-O-rutinoside; pmb0604, kaempferol 3-o-glucoside; pmb0618, 8-C-hexosyl-hesperetin O-hexoside; pmb0644, luteolin C-hexoside; pmb0696, 8-C-hexosyl chrysoeriol O-hexoside; pmb0745, tricin 4′-O-syringyl alcohol; pmb1466, tricin 4′-O-syringic acid; pmb2961, peonidin O-malonylhexoside; pmb2999, chrysoeriol 5-O-hexoside; pmb3045, tricin O-glucuronic acid; pme0197, quercetin 3-O-rutinoside; pme0324, chrysin; pme0359, apigenin 5-O-glucoside; pme0369, kaempferol 3-O-rutinoside; pme0431, procyanidin A1; pme1535, gallocatechin; pme1562, epicatechin gallate; pme2459, luteolin 7-O-glucoside; pme3250, biochanin A; pme3267, kaempferol 3-O-galactoside; pme3288, 3,7-di-O-methylquercetin; pme3300, tricetin.

Flavonoid accumulation and biosynthetic pathway in *C. paliurus* was constructed based on the transcriptomic and metabolomic data, KEGG pathway, and previous research (Figure 8). It mainly comprises 18 small branches, including Chrysin, Chrysoeriol, Tricin, Tricetin, and Naringenin. In the flaovnoids and anthocyanins biosynthetic pathways (pathway 00941), the expression level of the key structural genes, such as phenylalanine ammonialyase (PAL), cinnamate 4-hydroxylase (C4H), and chalcone synthase (CHS), are also shown in Figure 9. Notably, no significant expression of structural genes was found based on a threshold of the absolute fold change of log_2_ ≥ 1 with *q* ≤ 0.05 (Supplementary Table 7), indicating that the biosynthesis of flavonoids might not contribute to the different accumulation of flavonoids in the leaves of *C. paliurus* at different altitudes.

### Integrated Analysis of Metabolomic and Transcriptomic Data

**FIGURE 10:**
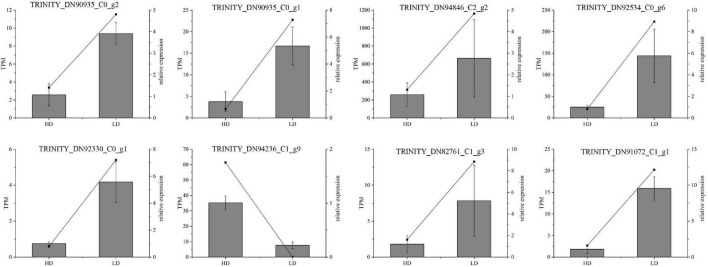
Real-time quantitative polymerase chain reaction (RT-qPCR) validation of candidate unigenes involved in *Cyclocarya paliurus* phenolic acid biosynthesis. The histogram shows the relative gene expression obtained via RT-qPCR. The transcripts per million (TPM) of each million mapped fragments of the transcriptome are represented by a line graph. The right *y*-axis indicates gene expression levels calculated as TPM. The left *y*-axis indicates relative gene expression levels obtained via RT-qPCR.

A total of 76 DAMs were significantly correlated with 791 DEGs at *p* < 0.01 and *r* > 0.9 (Supplementary Table 8). Among them, 31 flavonoids were significantly correlated with 227 DEGs, resulting in 412 related pairs (283 positive and 129 negative) between the DEGs and flavonoids (Figure 9 and Supplementary Table 8). According to the co-expression analysis of DEGs and DAMs (Figure 10), flavonoid accumulation was significantly positively correlated with the expression of various genes. For example, the accumulation of chrysoeriol O-glucuronic acid-O-hexoside (pmb0587) and TRINITY_DN92932_c0_g1, gallocatechin (pme1535) and TRINITY_DN85057_c3_g1, Epicatechin gallate (pme1562) and TRINITY_DN88894_c2_g1, TRINITY_DN97418_c1_g5, and TRINITY_DN86277_c2_g1, 3,7-di-O-methylquercetin (pme3288) and TRINITY_DN88894_c2_ g1, TRINITY_DN97418_c1_g5, and TRINITY_DN86277_c2_g1 was significantly correlated (Figure 7). However among the correlated DEGs, no gene annotated to known database was found (Supplementary Table 9). The accumulation of kaempferol 3-O-galactoside (pme3267) was significantly positively correlated with TRINITY_ DN94236_c1_g9 (Glyco_18 domain), TRINITY_DN84540_c1_g3, TRINITY_DN82915_c0_g5 (Ctr copper transporter family), TRINITY_ DN83542_c0_g1 (Cupin_1), and TRINITY_DN97420_c1_g1 (GH18_plant_chitinase_class_V) (Supplementary Table 9). The accumulation of Peonidin O-malonylhexoside (pmb2961) was significantly positively correlated with TRINITY_DN87410_c1_g2 (thiol-disulfide oxidoreductase), TRINITY_DN85057_c3_g1, TRINITY_DN88919_c2_g3 (phosphoglycerate kinase), and TRINITY_DN94391_c1_g2 (glycyl-tRNA synthetase) (Supplementary Table 9). The accumulation of chrysoeriol 5-O-hexoside (pmb2999) was significantly positively correlated with TRINITY_DN90591_c4_g6, TRINITY_DN94236_c1_g9 (Glyco_18 domain), TRINITY_ DN97420_c1_g12 (GH18_plant_chitinase_class_V), TRINITY_DN84518_c0_g5 (STKc_CAMK), TRINITY_DN86425_c1_g3 (rapid alkalinization factor 23-like protein), TRINITY_DN91096_ c2_g1 (adenylate kinase), TRINITY_DN86521_c2_g2 (Syntaxin-like protein), and TRINITY_DN84540_c1_g3 (Supplementary Table 9). However, no structural gene involved in flavonoid biosynthesis was found (Figure 8).

### Real-Time Quantitative PCR Validation of Gene Expression Profiles

The RT-qPCR assay was performed on 13 selected transcripts to further confirm the reliability and accuracy of the RNA-seq data. The results showed that the expression profiles of all the selected genes were similar to those detected by RNA-sequencing (Figure 10). Notably, the high *R*^2^ value indicates the high consistency between the two analyses (Figure 10).

## Discussion

### Effect of Altitude on Flavonoid Accumulation

Comparing metabolomic data obtained after analyzing leaf samples of *C. paliurus* collected at low and high altitude regions showed that 100 DAMs were higher at high altitude, while 46 were higher at low altitude. Furthermore, the contents of 66 flavonoids varied significantly between low and high altitudes. Among them, 47 were higher at high altitude, while only 19 were higher at low altitude. These results indicate that altitude significantly affects flavonoid accumulation, with more flavonoids accumulating at high altitude than at low altitude. Similar results were reported in other plants, such as P. kurroa (Kumari et al., 2020), and *H. pedunculosum* (Zhao et al., 2019). Zhao et al. (2019) found that tricetin in *H. pedunculosum* exhibited higher fold-change (83.85 ×) in high altitude than low altitude.

Flavonoids function as antioxidants by reducing DNA damage caused by reactive oxygen species accumulation. Naringin is a flavanone that exhibits antioxidant, antidiabetic, anti-dyslipidemic, and anti-inflammatory activities (Vinayagam and Xu, 2015; Nzuza et al., 2017). Tricetin is a potential anti-inflammatory and anticancer agent (Chang et al., 2017; Chung et al., 2017). Tricin showed anti-allergic (Lee et al., 2020) and anti-cancer (Li et al., 2021) activities. In this study, naringin was mainly found in the leaves collected at high altitude. Tricetin and tricin exhibited 2.53 and 3.50-fold increases at high altitude than at low altitudes, respectively. The levels of all the 11 tricin derivates increased at high altitude than at low altitude. Besides, tricin 7-O-β-guaiacylglycerol and tricin O-malonyl rhamnoside were almost only found in the leaves collected at high altitude., These results indicate that *C. paliurus* grown at high altitude exhibit high flavonoid accumulation, thus potentially exerting higher hypoglycemic, antioxidant, anti-allergic, and anti-cancer activities than those grown at low altitude.

### Effect of Altitude on Flavonoid Biosynthesis

Most studies have focused on integrating flavonoid accumulation and biosynthesis processes. However, there is limited research on the effect of altitude on flavonoid biosynthesis. Wen and Wang (2003) found that high altitude is likely to accelerate mutation events related to flavonoid biosynthesis. Sheng et al. (2021) analyzed flavonoid biosynthesis by sampling the leaves at different developmental stages and found that the expression of most of the structural genes was positively related to flavonoid accumulation. For example, the accumulation of tricin 7-O-hexoside, tricin 7-O-feruloylhexoside, and kaempferol 3-O-rhamnoside (kaempferin) were significantly positively correlated with the expression of PAL-1 and UGT72E-2. Also, the accumulation trends of kaempferol and delphinidin were highly similar to the expression trends of FLS-1, FLS-2, and DFR genes (Sheng et al., 2021). In this study, integrated analysis of transcriptomic and metabolomic data indicated that several DEGs were positively correlated with DAMs (283 pairs). However, these genes were not among the structural genes identified previously to participate in flavonoid biosynthetic pathway, such as flavanone 3-hydroxylase (F3H), flavanone 4-reductase (DFR), and flavonol synthase (FLS) genes (Luo et al., 2016; Shen et al., 2020; Sheng et al., 2021). The lack of annotation involved with the structural genes involved in the biosynthesis of flavonoids indicated that the involved DEGs might not contribute to the biosynthesis of flavonoids, but related with the transport and accumulation of flavonoids.

Another possible reason for the lack of relationship between the structural genes and flavonoid biosynthesis might be due to the developmental stage of the plants at the time the leaves were collected. Specifically, the matured leaves at F4 stage analyzed in the present study could have passed the flavonoid biosynthesis stage, thus the structural genes related to flavonoid biosynthesis might have been suppressed or down-regulated (Sheng et al., 2021). Specifically, Sheng et al. (2021) found that flavonoid contents at F4 stage were lower than those at F3 stage. Yin et al. (2015) found that anthocyanin biosynthesis genes were up-regulated in the leaves at the early stages of growth, indicating that flavonoid biosynthesis might vary with the age of plants and might be suppressed or down-regulated at the later stages of growth. Thus, further studies should integrate the developmental factors into the study and compare the effect of altitude on the leaves sampled at different developmental stages.

### Possible Mechanisms of Flavonoid Accumulation at High Altitude

High altitude is typically characterized by harsh environmental conditions, including increased UV-radiation (Reyes et al., 2020), low temperature, low oxygen, and reduced pathogen incidence (Zhang et al., 2019). Certain secondary metabolites have been shown to increase at high altitudes due to the induction by the harsh environmental stress (Zhang et al., 2019; Kumari et al., 2020; Yu et al., 2021). Flavonoid accumulation is induced by abiotic stresses, such as extreme temperatures, UV radiation, and drought, and is crucial for species adapting to extreme environments (Dong and Lin, 2021; Zhang et al., 2021). Tricetin plays an essential role in plant tolerance to biotic/abiotic stresses (Agharbaoui et al., 2015; Zhao et al., 2019). The accumulation of tricetin’s major product (tricin) has been shown to occur at different stages of wheat development following exposure to different abiotic stresses such as cold, salt, and drought (Moheb et al., 2013). Base excision repair is a vital genome maintenance pathway that eliminates endogenously damaged DNA bases from cells to reduce their accumulation. These DNA damage are induced by the attack of oxidants, alkylating agents, ultraviolet (UV) light, and other forms of electromagnetic radiation (Fromme and Verdine, 2004; Marsden et al., 2017). In this study, base excision repair is one of the KEGG categories mapped by the 91 up-regulated DEGs (Supplementary Figure 2). The up-regulation of DEGs involved in base excision repair indicates that *C. paliurus* at high altitude potentially undergo changes in the expression of specific genes related to the removal of damaged DNA bases from cells. There gene expression changes are possibly induced by high UV-light, which is the harshest environmental factor at high altitudes.

Metabolite accumulation in plants might be due to coordinated remobilization and relocation of metabolites between source and sink organs (Reyes et al., 2020). Thus, variation in flavonoid accumulation in the leaves of *C. paliurus* between high and low altitude might result from changes in flavonoid different transport in response to harsh environmental factors. Among the 227 DEGs identified in this study, 101 were successfully annotated according to the known protein database CDD (Supplementary Table 9). Although they are not the structural genes involved in flavonoid biosynthesis, they are implicated involved in energy and protein synthesis. TRINITY_DN91096_c2_g1 was annotated as adenylate kinase. Adenylate kinase is a ubiquitous and abundant enzyme catalyzing phosphoryl transfer between two adenosine diphosphate (ADP) molecules to yield adenosine triphosphate (ATP) and adenosine monophosphate (AMP) (Feher, 2012). TRINITY_DN88919_c2_g3 was annotated as phosphoglycerate kinase. Phosphoglycerate kinase is an enzyme that facilitates glycolysis. It catalyzes high-energy phosphoryl transfer of the acyl phosphate of 1,3-bisphosphoglycerate to ADP to produce ATP (Blake and Rice, 1981). ATP is a common energy source for synthetic, mechanical, and transport activities (Feher, 2012). Biosynthesis of the secondary metabolites is an energy-consuming process (Wang et al., 2002). Of note, transportation of flavonoids is also an energy-consuming process. Thus, there might be crosstalk between adenylate kinase and flavonoid accumulation. ATP-binding cassette transporters were implicated in the sequestration of flavonoids into the vacuole (Zhao and Dixon, 2010; Petrussa et al., 2013). ATP synthesis induced at high altitude by multiple gene expressions potentially contributes to the accelerated transportation of flavonoids into the vacuole in the leaves of *C. paliurus*.

Also, TRINITY_DN87410_c1_g2 was annotated as thiol-disulfide oxidoreductase. Thiol-disulfide oxidoreductases facilitate disulfide bond formation in proteins that are exported from the cytoplasm (Dorenbos et al., 2002). TRINITY_DN94391_c1_g2 was annotated as the glycyl-tRNA synthetase. Glycyl–tRNA synthetase facilitate glycine attachment to tRNAs, which is essential for protein synthesis. Increased activities of various eynzymes and other proteins, and the consequent increase in energy levels (ATP) might contribute to flavonoid transport into the vacuoles in the leaves of *C. paliurus*.

### Trade-off Hypothesis

The hypothesis of a contrasting plant strategy suggest a trade-off between plant biomass production and the accumulation of secondary metabolites, such as flavonoids (Hofmann and Jahufer, 2011). Notably, the findings of this study support the trade-off hypothesis. For example, although 100 DAMs increased at high altitude, 641 DEGs were down-regulated in the leaves at high altitude, while only 91 were up-regulated at low altitude. A total of 641 down-regulated DEGs were mapped onto “Oxidative phosphorylation” followed by subcategory “cell cycle” and “splicesome,” etc. (Supplementary Figure 3). Oxidative phosphorylation plays a central role in sugar metabolism by supplying the carbon skeleton and motive force for biochemical reactions (Wang et al., 2017). Cell cycle is directly related to cell growth (Zyskind and Smith, 1992). Splicesome participates in the process of pre-mRNA splicing (Matera and Wang, 2014). The down-regulated DEGs might be related to the reduced biomass accumulation in the leaves of *C. paliurus*. Except for base excision repair category, the 91 up-regulated DEGs were also mapped to photosynthesis, cell cycle, and oxidative phosphorylation (Supplementary Figure 2). The products of photosynthesis might act as substrates for the production of secondary metabolites (Mi et al., 2009). The up-regulated DEGs involved in photosynthesis, cell cycle, and oxidative phosphorylation are possibly feedback responses to the reduced biomass accumulation in the leaves, induced by harsh environmental factors at high altitudes, such as low temperature and oxygen.

## Conclusion

This study shows that high altitude induces more flavonoid accumulation in *C. paliurus* leaves than low altitude. This possibly occurs due to the responses of *C. paliurus* to harsh environmental factors at high altitude. Notably, high UV-light might be the main environmental factor influencing flavonoid accumulation in high altitudes. The possible mechanisms underlying the differentially accumulation of flavonoids at different altitudes might involve changes in transport and relocation of flavonoids in *C. paliurus* leaves, but not variations in flavonoid biosynthetic pathways. The up-regulation of genes related to energy and protein synthesis, but not the expression of the structural genes involved in flavonoid biosynthesis, potentially contribute to flavonoid accumulation at high altitudes. However, future studies should focus on uncovering the functions of the candidate DEGs to precisely identify the main gene influencing flavonoid biosynthesis and accumulation at high altitudes. These results broaden our understanding of how altitude affects metabolite biosynthesis in plants. Further studies are needed to fully elucidate the functions of flavonoid biosynthesis-related genes and their possible pathways.

## Data Availability Statement

The original contributions presented in the study are publicly available. This data can be found here: National Center for Biotechnology Information (NCBI) BioProject database under accession number PRJNA769609.

## Author Contributions

JL designed the research. ZD, WL, and BY performed the research and analyzed the data. ZD and WL wrote the first draft of the manuscript. All authors commented on previous versions of the manuscript, read, and approved the final manuscript.

## Conflict of Interest

The authors declare that the research was conducted in the absence of any commercial or financial relationships that could be construed as a potential conflict of interest.

## Publisher’s Note

All claims expressed in this article are solely those of the authors and do not necessarily represent those of their affiliated organizations, or those of the publisher, the editors and the reviewers. Any product that may be evaluated in this article, or claim that may be made by its manufacturer, is not guaranteed or endorsed by the publisher.

## Footnotes

1
http://www.kegg.jp/kegg/compound/

2
http://www.kegg.jp/kegg/pathway.html

## Supplementary Material

Supplementary Figure 1 Leaves of *Cyclocarya paliurus* grown at high altitude (HA) and low altitude (LA). Scale bar in the left top was 1 cm.

Supplementary Figure 2 KEGG pathway terms assignment of 91 up-regulated genes in the leaves of *Cyclocarya paliurus* at high altitude than at low altitude.

Supplementary Figure 3 KEGG pathway terms assignment of 641 down-regulated genes in the leaves of *Cyclocarya paliurus* at high altitude than at low altitude.

## References

[B1] Agharbaoui Z. Leclercq M. Remita M. A. Badawi M. A. Lord E. Houde M. (2015). An integrative approach to identify hexaploid wheat miRNAome associated with development and tolerance to abiotic stress. *BMC Genomics* 16:339. 10.1186/s12864-015-1490-8 25903161 PMC4443513

[B2] Blake C. C. F. Rice D. (1981). Phosphoglycerate kinase. *Philos. Trans. R. Soc. B Biol. Sci.* 293 93–104. 10.1098/rstb.1981.0063 6115427

[B3] Chang P. Y. Hsieh M. J. Hsieh Y. S. Chen P. N. Yang J. S. Lo F. C. (2017). Tricetin inhibits human osteosarcoma cells metastasis by transcriptionally repressing MMP-9 via p38 and Akt pathways. *Environ. Toxicol.* 32 2032–2040. 10.1002/tox.22380 27860196

[B4] Chung T. T. Chuang C. Y. Teng Y. H. Hsieh M. J. Lai J. C. Chuang Y. T. (2017). Tricetin suppresses human oral cancer cell migration by reducing matrix metalloproteinase-9 expression through the mitogen-activated protein knase signaling pathway. *Environ. Toxicol.* 32 2392–2399. 10.1002/tox.22452 28731287

[B5] Dong N. Q. Lin H. X. (2021). Contribution of phenylpropanoid metabolism to plant development and plant-environment interactions. *J. Integr. Plant Biol.* 63 180–209. 10.1111/jipb.13054 33325112

[B6] Dorenbos R. Stein T. Kabel J. Bruand C. Bolhuis A. Bron S. (2002). Thiol-disulfide oxidoreductases are essential for the production of the lantibiotic sublancin 168. *J. Biol. Chem.* 277 16682–16688. 10.1074/jbc.M201158200 11872755

[B7] Feher J. (2012). *ATP production I: glycolysis Quantitative Human Physiology, An introduction.* London: Academic Press, 171–179. 10.1016/B978-0-12-382163-8.00020-7

[B8] Fromme J. C. Verdine G. L. (2004). Base excision repair. *Adv. Protein Chem.* 69 1–41. 10.1016/S0065-3233(04)69001-2 15588838

[B9] Hao Y. Xiong Y. Cheng Y. L. Song G. Jia C. X. Qu Y. H. (2019). Comparative transcriptomics of 3 high-altitude passerine birds and their low-altitude relatives. *Proc. Natl. Acad. Sci. U. S. A.* 116 11851–11856. 10.1073/pnas.1819657116 31127049 PMC6576129

[B10] Hofmann R. W. Jahufer M. Z. Z. (2011). Tradeoff between biomass and flavonoid accumulation in white clover reflects contrasting plant strategies. *PLoS One* 6:e18949. 10.1371/journal.pone.0018949 21526153 PMC3079752

[B11] Kumari M. Joshi R. Kumar R. (2020). Metabolic signatures provide novel insights to *Picrorhiza kurroa* adaptation along the altitude in Himalayan region. *Metabolomics* 17:77. 10.1007/s11306-020-01698-8 32577832

[B12] Lee J. Y. Park S. H. Jhee K. H. Yang S. A. (2020). Tricin isolated from enzyme-treated *Zizania latifolia* extract inhibits IgE-mediated allergic reactions in RBL-2H3 cells by targeting the Lyn/Syk pathway. *Molecules* 25:2084. 10.3390/molecules25092084 32365709 PMC7249134

[B13] Li J. X. Li R. Z. Sun A. Zhou H. Neher E. Yang J. S. (2021). Metabolomics and integrated network pharmacology analysis reveal tricin as the active anti-cancer component of Weijing decoction by suppression of PRKCA and sphingolipid signaling. *Pharmacol. Res.* 171:105574. 10.1016/j.phrs.2021.105574 34419228

[B14] Lin W. D. Chen H. W. Wang J. M. Zheng Y. L. Lu Q. W. Zhu Z. P. (2021). Transcriptome analysis associated with polysaccharide synthesis and their antioxidant activity in *Cyclocarya paliurus* leaves of different developmental stages. *Peer J* 9:e11615. 10.7717/peerj.11615 34178473 PMC8210810

[B15] Lin W. D. Li Y. L. Lu Q. W. Lu H. F. Li J. M. (2020). Combined analysis of the metabolome and transcriptome identified candidate genes involved in phenolic acid biosynthesis in the leaves of *Cyclocarya paliurus*. *Int. J. Mol. Sci.* 21:1337. 10.3390/ijms21041337 32079236 PMC7073005

[B16] Liu Y. Fang S. Z. Zhou M. M. Shang X. L. Yang W. X. Fu X. X. (2018). Geographic variation in water-soluble polysaccharide content and antioxidant activities of *Cyclocarya paliurus* leaves. *Ind. Crops Prod.* 121 180–186. 10.1016/j.indcrop.2018.05.017

[B17] Liu Y. J. Zhao H. Y. Luo Q. H. Yang Y. D. Zhang G. S. Zhou Z. Y. (2020). De novo transcriptomic and metabolomic analyses reveal the ecological adaptation of high-altitude *Bombus pyrosoma*. *Insects* 11:631. 10.3390/insects11090631 32937786 PMC7563474

[B18] Luo P. Ning G. G. Wang Z. Shen Y. X. Jin H. A. Li P. H. (2016). Disequilibrium of flavonol synthase and dihydroflavonol-4-Reductase expression associated tightly to white vs. red color flower formation in plants. *Front. Plant Sci.* 6:1257. 10.3389/fpls.2015.01257 26793227 PMC4710699

[B19] Ma L. Sun X. D. Kong X. X. Galvan J. V. Li X. Yang S. H. (2015). Physiological, biochemical and proteomics analysis reveals the adaptation strategies of the alpine plant *Potentialla saundersiana* at altitude gradient of the Northewestern Tibetan Plateau. *J. Proteom.* 112 63–82. 10.1016/j.jprot.2014.08.009 25181701

[B20] Marsden C. G. Dragon J. A. Wallace S. S. Sweasy J. B. (2017). Base excision repair variants in cancer. *Methods Enzymol.* 591 119–157. 10.1016/bs.mie.2017.03.003 28645367 PMC5859333

[B21] Matera A. G. Wang Z. (2014). A day in the life of the spliceosome. *Nat. Rev. Mol. Cell. Bio.* 15 108–121. 10.1038/nrm3742 24452469 PMC4060434

[B22] Mi L. X. Shangguan X. C. Shi L. X. Yin Z. P. Zhou B. Y. (2009). Studies on the determination and distribution of Total flavonoids of *Cyclocarya paliurus* vegetative organs. *Acta Agric. Univ. Jiangxiensis* 31 896–900.

[B23] Mo T. Y. Meng T. Meng F. (2014). Distribution and application of *Cyclocarya paliurus* resources in Guiling region. *South China Agric.* 18 71–73.

[B24] Moheb A. Agharbaoui Z. Kanapathy F. Ibrahim R. K. Roy R. Sarhan F. (2013). Tricin biosynthesis during growth of wheat under different abiotic stresses. *Plant Sci.* 20 115–120. 10.1016/j.plantsci.2012.12.005 23352409

[B25] Nzuza S. Zondi S. Owira P. M. O. (2017). Naringin prevents HIV-1 protease inhibitors-induced metabolic complications in vivo. *PLoS One* 12:e0183355. 10.1371/journal.pone.0183355 29121676 PMC5679664

[B26] Petrussa E. Braidot E. Zancani M. Peresson C. Bertolini A. Patui S. (2013). Plant flavonoids-biosynthesis, transport and involvement in stress responses. *Int. J. Mol. Sci.* 14 14950–14973. 10.3390/ijms140714950 23867610 PMC3742282

[B27] Reyes T. H. Esparza E. Crestani G. Limonchi F. Cruz R. Salinas N. (2020). Physiological response of maca (*Lepidium meyenii* Walp.) plants to UV radiation in its high-altitude mountain ecosystem. *Sci. Rep.* 10:2654. 10.1038/s41598-020-59638-4 32060345 PMC7021813

[B28] Shen J. C. Shao W. L. Du Z. K. Lu H. F. Li J. M. (2020). Integrated metabolomic and transcriptomic analyses reveal differences in the biosynthetic pathway of anthocyanins in *Fragaria nilgerrensis* and *Fragaria pentaphylla*. *Sci. Hortic.* 271:109476. 10.1016/j.scienta.2020.109476

[B29] Sheng X. L. Chen H. W. Wang J. M. Zheng Y. L. Li Y. L. Jin Z. X. (2021). Joint transcriptomic and metabolic analysis of flavonoids in *Cyclocarya paliurus* leaves. *ACS Omega* 6 9028–9038. 10.1021/acsomega.1c00059 33842773 PMC8028134

[B30] Tang Q. Z. Gu Y. R. Zhou X. M. Jin L. Guan J. Q. Liu R. (2017). Comparative transcriptomics of 5 high-altitude vertebrates and their low-altitude relatives. *GigaScience* 6 1–9. 10.1093/gigascience/gix105 29149296 PMC5729692

[B31] Vinayagam R. Xu B. (2015). Antidiabetic properties of dietary flavonoids: a cellular mechanism review. *Nutr. Metab.* 12:60. 10.1186/s12986-015-0057-7 26705405 PMC4690284

[B32] Voronkov A. S. Ivanova T. V. Kuznetsova E. I. Kumachova T. K. (2019). Adaptations of malus domestica borkh. (*rosaceae*) fruits grown at different altitudes. *Russ. J. Plant Physiol.* 66 922–931. 10.1134/S1021443719060153

[B33] Wang H. F. Ju X. R. He G. B. Jin X. Q. Chen J. (2002). Effects of altitudes and seasons on the flavonoid content of Ginkgo biloba leaves. *Chem. Ind. For. Prod.* 22 47–50.

[B34] Wang Q. H. Zhao C. Zhang M. Li Y. Z. Shen Y. Y. Guo J. X. (2017). Transcriptome analysis around the onset of strawberry fruit ripening uncovers an important role of oxidative phosphorylation in ripening. *Sci. Rep.* 7:41477. 10.1038/srep41477 28195221 PMC5307319

[B35] Wang X. Y. Lin S. H. Zhang Z. F. (2020). Comparison of active components and biological activities of *Cyclocarya paliurus* at different altitudes. *Zhejiang Agric. Sci.* 61 2545–2547.

[B36] Wen D. B. Wang H. F. (2003). Mutation effect of high altitude environment on the synthesis of flavonoids in the inflorescence of *Celosia cristata* L. *Chin. Acad. Med. Mag. Organ.* 2 26–31.

[B37] Wu X. X. (2019). *Study on plus tree selection of Cyclocarya paliurus and analysis of natural forest site conditions.* Ph.D. thesis. China: Anhui Agricultural University

[B38] Yang H. M. Yin Z. Q. Zhao M. G. Jiang C. H. Zhang J. Pan K. (2018). Pentacyclic triterpenoids from *Cyclocarya paliurus* and their antioxidant activities in FFA-induced HepG2 steatosis cells. *Phytochemistry* 151 119–127. 10.1016/j.phytochem.2018.03.010 29679877

[B39] Yin J. M. Yan R. X. Zhang P. T. Han X. Y. Wang L. (2015). Anthocyanin accumulation rate and the biosynthesis related gene expression in *dioscorea alata*. *Biol. Plant.* 59 325–330. 10.1007/s10535-015-0502-5

[B40] Yu J. Q. Song T. T. He J. L. Kongling Z. L. Lian L. He W. H. (2021). Integrated physiological and transcriptomic analyses responses to altitude stress in Oat (*Avena sativa* L.). *Front. Genet.* 12:638683. 10.3389/fgene.2021.638683 34220929 PMC8248544

[B42] Zhang T. Qiao Q. Novikova P. Y. Wang Q. Yue J. Guan Y. L. (2019). Genome of *Crucihimalaya himalaica*, a close relative of Arabidopsis, shows ecological adaptation to high altitude. *Proc. Natl. Acad. Sci. U. S. A.* 116 7137–7146. 10.1073/pnas.1817580116 30894495 PMC6452661

[B43] Zhang X. Sun Y. X. Landis J. B. Shen J. Zhang H. J. Kuang T. H. (2021). Transcriptomes of Saussurea (*Asteraceae*) provide insights into high-altitude adaptation. *Plants* 10:1715. 10.3390/plants10081715 34451759 PMC8402177

[B44] Zhang Y. T. Li Y. L. He Y. W. Hu W. J. Zhang Y. Wang X. R. (2018). Identification of NADPH oxidase family members associated with cold stress in strawberry. *FEBS Open Biol.* 8 593–605. 10.1002/2211-5463.12393 29632812 PMC5881550

[B45] Zhao J. Dixon R. A. (2010). The ‘ins’ and ‘outs’ of flavonoid transport. *Trends Plant Sci.* 15 72–80. 10.1016/j.tplants.2009.11.006 20006535

[B46] Zhao S. Zhang X. Su Y. Chen Y. Liu Y. Sun M. (2018). Transcriptome analysis reveals dynamic fat accumulation in the walnut kernel. *Int. J. Genomics* 2018:8931651. 10.1155/2018/8931651 30622952 PMC6304212

[B47] Zhao Y. Xu F. L. Liu J. Guan F. C. Quan H. Meng F. J. (2019). The adaptation strategies of *Herpetospermum pedunculosum* (Ser.) Baill at the latitude gradient of the Tibetan plateau by physiological and metabolomic methods. *BMC Genomics* 20:451. 10.1186/s12864-019-5778-y 31159723 PMC6547600

[B48] Zhou M. Lin Y. Fang S. Liu Y. Shang X. (2019b). Phytochemical content and antioxidant activity in aqueous extracts of *Cyclocarya paliurus* leaves collected from different populations. *Peer J* 7:e6492. 10.7717/peerj.6492 30809459 PMC6385679

[B49] Zhou M. Chen P. Lin Y. Fang S. Shang X. (2019a). A comprehensive assessment of bioactive metabolites, antioxidant and antiproliferative activities of *Cyclocarya paliurus* (Batal.) Iljinskaja leaves. *Forests* 10:625. 10.3390/f10080625

[B50] Zyskind J. W. Smith D. W. (1992). DNA replication, the bacterial cell cycle, and cell growth. *Cell* 69 5–8. 10.1016/0092-8674(92)90112-P 1555241

