# Supplementary figures and images for: Integrated Metabolomic and Transcriptomic Analysis of the Flavonoid Accumulation in the Leaves of Cyclocarya paliurus at Different Altitudes
Source: Front Plant Sci. 2022 Feb 8;12:794137. doi: 10.3389/fpls.2021.794137 (PMC8860981; doi:10.3389/fpls.2021.794137)

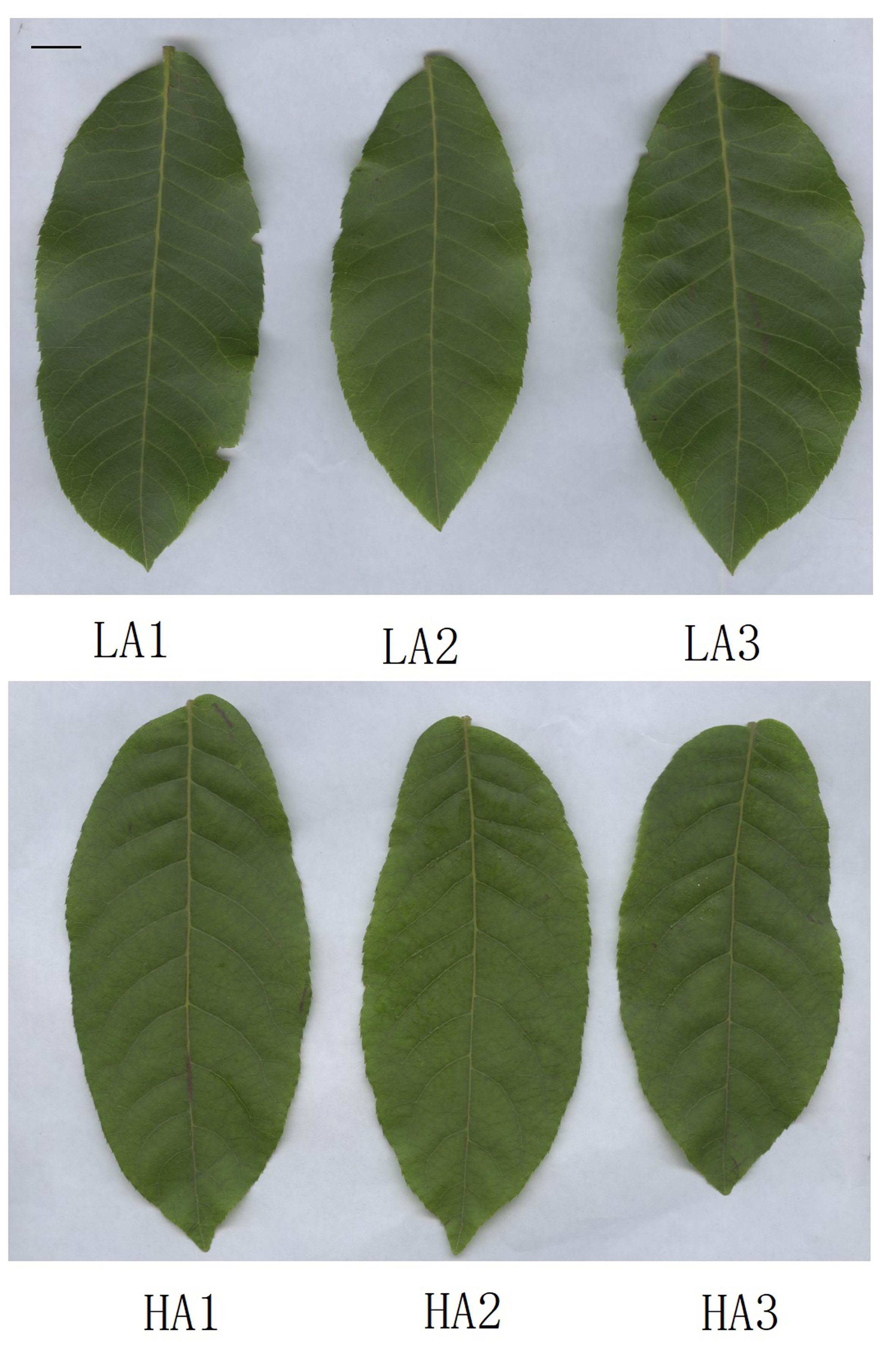

Supplement: Supplementary Figure 1 — Leaves of Cyclocarya paliurus grown at high altitude (HA) and low altitude (LA). Scale bar in the left top was 1 cm. [file Image_1.JPEG]

# KEGG Enrichment

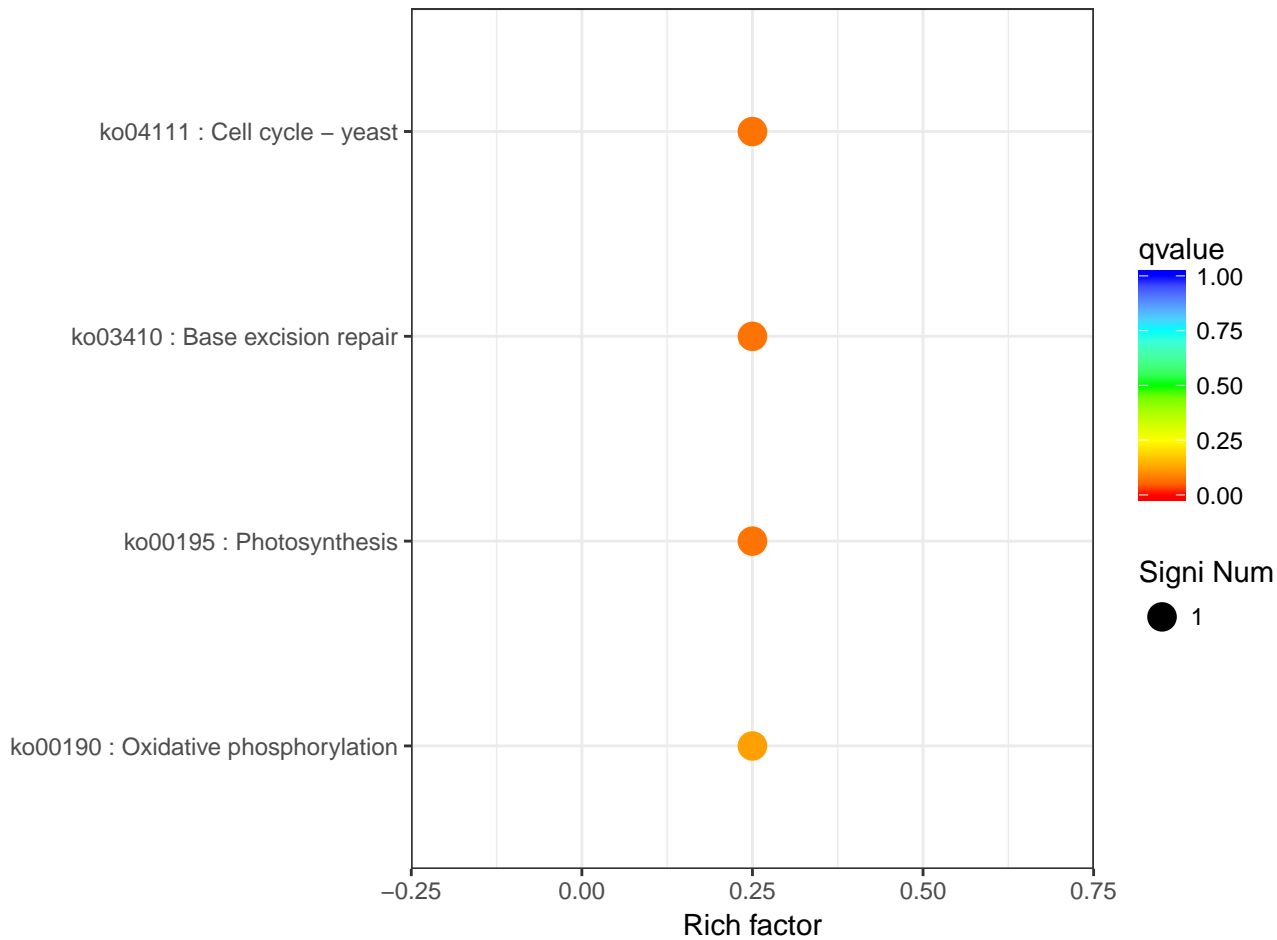

Supplement: Supplementary Figure 2 — KEGG pathway terms assignment of 91 up-regulated genes in the leaves of Cyclocarya paliurus at high altitude than at low altitude. [file Image_2.pdf]

# KEGG Enrichment

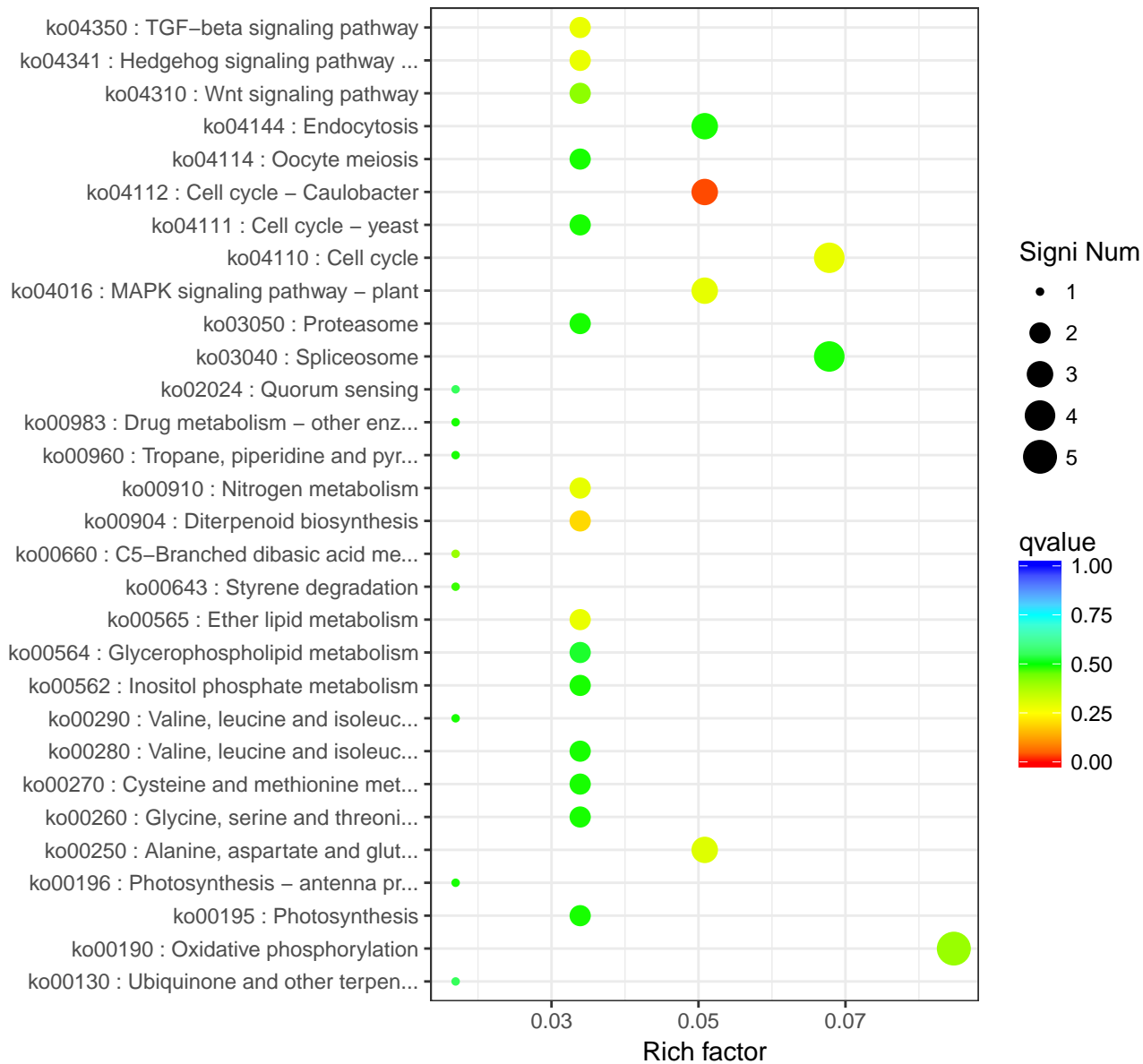

Supplement: Supplementary Figure 3 — KEGG pathway terms assignment of 641 down-regulated genes in the leaves of Cyclocarya paliurus at high altitude than at low altitude. [file Image_3.pdf]
