# Supplementary material for: Integrated Metabolomic and Transcriptomic Analysis of the Flavonoid Accumulation in the Leaves of Cyclocarya paliurus at Different Altitudes
Source: Front Plant Sci. 2022 Feb 8;12:794137. doi: 10.3389/fpls.2021.794137 (PMC8860981; doi:10.3389/fpls.2021.794137)
Supplement: Supplementary file 5 [file Table_2.docx]

Table S2 The characteristics of the leaves collected at different altitude.

| Altitude | Area (cm^2^) | Width (cm) | Length (cm) | Perimeter (cm) |
| --- | --- | --- | --- | --- |
| Low | 31.64±4.50a | 4.62±0.38a | 9.55±0.90a | 25.89±2.44a |
| High | 35.28±4.79b | 4.72±0.31a | 10.29±1.00b | 27.07±2.11a |
