# Supplementary material for: Integrated Metabolomic and Transcriptomic Analysis of the Flavonoid Accumulation in the Leaves of Cyclocarya paliurus at Different Altitudes
Source: Front Plant Sci. 2022 Feb 8;12:794137. doi: 10.3389/fpls.2021.794137 (PMC8860981; doi:10.3389/fpls.2021.794137)
Supplement: Supplementary file 6 [file Table_3.docx]

Table S3. The primers used for real-time quantitative polymerase chain reaction

| NO | Gene ID | Sense primer （SP） | Anti-sense primer （AP） |
| --- | --- | --- | --- |
| 1 | TRINITY_DN94846_c2_g2 | 5'-GAACCTTGCCGAGATGTAT-3' | 5'-GCCACTCTTCCTAATAACCT-3' |
| 2 | TRINITY_DN90935_c0_g2 | 5'-TCGTCGGAGGCAATAGTT-3' | 5'-CGTCTCTGTACCACTCAAG-3' |
| 3 | TRINITY_DN90935_c0_g1 | 5'-CTCATCATTCTCGGATTCAAG-3' | 5'-GCCTGCGTTATTGCCTTA-3' |
| 4 | TRINITY_DN92534_c0_g6 | 5'-CCACATCCAACATCAACTAC-3' | 5'-TCACTCTGCTTCCTTCATC-3' |
| 5 | TRINITY_DN82761_c1_g3 | 5'-CAACGGCACGATGATATAC-3' | 5'-ACGAGAAGGTAATGAACTGT-3' |
| 6 | TRINITY_DN91072_c1_g1 | 5'-GGCTCAATCTCCTTAACTATG-3' | 5'-TCTCTTCTGTCTCCTCCAA-3' |
| 7 | TRINITY_DN92330_c0_g1 | 5'-TCTTCGTCCTCCTCATCTT-3' | 5'-AGCGTTAATAGTGGCAGTAA-3' |
| 8 | TRINITY_DN94236_c1_g9 | 5'-CCAATAAGGTGCGGAAGT-3' | 5'-GCTACATCCATCATCATAACC-3' |
| 9 | *β-actin-1* | 5'-CTCTTCCAGCCATCCATGATCG-3' | 5'-CCACTGAGGACAATATTGCCAT-3' |
