# Supplementary material for: Integrated Metabolomic and Transcriptomic Analysis of the Flavonoid Accumulation in the Leaves of Cyclocarya paliurus at Different Altitudes
Source: Front Plant Sci. 2022 Feb 8;12:794137. doi: 10.3389/fpls.2021.794137 (PMC8860981; doi:10.3389/fpls.2021.794137)
Supplement: Supplementary file 8 [file Table_5.docx]

Table S5. Differentially accumulated flavonoids in the leaves of *Cyclocarya paliurus* plants growing at low and high altitudes

| Index | Compounds | High altitude | Low altitude | VIP | High altitude/Low altitude |
| --- | --- | --- | --- | --- | --- |
| **Chrysin and its derivatives** | | | | | |
| pma0825 | Chrysin O-malonylhexoside | 13103.00 | 114643.3333 | 2.16 | 0.11 |
| pme0324 | Chrysin | 34000.00 | 73300 | 1.18 | 0.46 |
| pme2979 | Pinocembrin (Dihydrochrysin) | 129733.33 | 299666.6667 | 1.27 | 0.43 |
| **Apigenin derivatives** | | | | | |
| pma1108 | Apigenin C-glucoside | 20800000.00 | 5330000 | 1.65 | 3.90 |
| pmb0672 | 6-C-hexosyl-apigenin O-feruloylhexoside | 14406.00 | 74000 | 3.15 | 0.19 |
| pme0359 | Apigenin 5-O-glucoside | 1060333.33 | 258669.6667 | 2.33 | 4.10 |
| pme0368 | Apigenin 7-rutinoside (Isorhoifolin) | 257003.00 | 58339.33333 | 1.71 | 4.41 |
| **Chrysoeriol and its derivatives** | | | | | |
| pma0253 | O-methylChrysoeriol 5-O-hexoside | 10193333.33 | 884000 | 1.84 | 11.53 |
| pma6638 | O-methylChrysoeriol 7-O-hexoside | 10670000.00 | 953333.3333 | 1.81 | 11.19 |
| pmb0587 | Chrysoeriol O-glucuronic acid-O-hexoside | 313000.00 | 1245666.667 | 1.58 | 0.25 |
| pmb0600 | Chrysoeriol 7-O-rutinoside | 52669.67 | 9 | 3.05 | 5852.19 |
| pmb0696 | 8-C-hexosyl chrysoeriol O-hexoside | 80003.00 | 273333.3333 | 2.13 | 0.29 |
| pmb0701 | Chrysoeriol 8-C-hexoside | 676000.00 | 138366.6667 | 1.70 | 4.89 |
| pmb2999 | Chrysoeriol 5-O-hexoside | 2050000.00 | 6583333.333 | 1.53 | 0.31 |
| pme0363 | Chrysoeriol | 8876666.67 | 2583333.333 | 1.48 | 3.44 |
| **Kaempferol derivatives** | | | | | |
| pmb0604 | Kaempferol 3-O-glucoside (Astragalin) | 2246666.67 | 12206666.67 | 1.68 | 0.18 |
| pme0369 | Kaempferol 3-O-rutinoside (Nicotiflorin) | 514333.33 | 94500 | 1.65 | 5.44 |
| pme1605 | Kaempferol 3-O-robinobioside (Biorobin) | 487000.00 | 66700 | 1.82 | 7.30 |
| pme3267 | Kaempferol 3-O-galactoside (Trifolin) | 102933.33 | 493333.3333 | 1.60 | 0.21 |
| pma1116 | Kaempferide | 1140333.33 | 260000 | 1.75 | 4.39 |
| **Luteolin derivatives** | | | | | |
| pma6496 | Luteolin 6-C-glucoside | 2361666.67 | 616000 | 1.48 | 3.83 |
| pmb0644 | Luteolin C-hexoside | 2649666.67 | 649666.6667 | 1.57 | 4.08 |
| pmb0665 | Luteolin 8-C-hexosyl-O-hexoside | 351666.67 | 80533.33333 | 1.72 | 4.37 |
| pme2459 | Luteolin 7-O-glucoside (Cynaroside) | 32466666.67 | 167266666.7 | 1.71 | 0.19 |
| **Naringenin derivatives** | | | | | |
| pma0724 | Naringenin C-hexoside | 1185000.00 | 392333.3333 | 1.33 | 3.02 |
| pma0791 | Naringenin O-malonylhexoside | 29869.67 | 111466.6667 | 1.94 | 0.27 |
| pme0330 | Naringenin 7-O-neohesperidoside (Naringin) | 57333.33 | 9 | 4.32 | 6370.37 |
| **Quercetin derivatives** | | | | | |
| pma0214 | methylQuercetin O-hexoside | 599000.00 | 107000 | 1.47 | 5.60 |
| pmb0711 | Quercetin 7-O-rutinoside | 201000.00 | 47833.33333 | 1.66 | 4.20 |
| pme0197 | Quercetin 3-O-rutinoside (Rutin) | 1381000.00 | 322666.6667 | 1.63 | 4.28 |
| pme2977 | Troxerutin (Trihydroxyethyl rutin) | 82530.00 | 5346.666667 | 1.65 | 15.44 |
| pme3288 | 3,7-Di-O-methylquercetin | 715333.33 | 105833.3333 | 1.65 | 6.76 |
| **Tricin and its derivatives** | | | | | |
| pmb0724 | Tricin O-rhamnoside | 4036666.67 | 498800 | 2.13 | 8.09 |
| pmb0725 | Tricin 7-O-feruloylhexoside | 292666.67 | 31603 | 2.54 | 9.26 |
| pmb0738 | Tricin O-sinapoylhexoside | 332900.00 | 20103 | 2.59 | 16.56 |
| pmb0743 | Tricin 7-O-β-guaiacylglycerol | 12033.33 | 9 | 3.96 | 1337.04 |
| pmb0745 | Tricin 4'-O-syringyl alcohol | 14300.00 | 6303 | 1.73 | 2.27 |
| pmb0746 | Tricin 4'-O-β-guaiacylglycerol | 1294000.00 | 383000 | 1.18 | 3.38 |
| pmb1312 | Tricin 4'-O-(β-guaiacylglyceryl) ether 7-O-hexoside | 195666.67 | 73033.33333 | 1.35 | 2.68 |
| pmb1466 | Tricin 4'-O-syringic acid | 109166.67 | 15869.66667 | 2.23 | 6.88 |
| pmb2850 | Tricin | 19756666.67 | 5644333.333 | 1.51 | 3.50 |
| pmb3032 | Tricin O-malonyl rhamnoside | 6660.00 | 9 | 3.73 | 740.00 |
| pmb3045 | Tricin O-glucuronic acid | 29433.33 | 13703 | 1.76 | 2.15 |
| pmb3047 | Tricin 4'-O-(syringyl alcohol) ether 5-O-hexoside | 76500.00 | 27133.33333 | 1.31 | 2.82 |
| **Myricetin derivatives** | | | | | |
| pme1551 | Myricetin 3-O-rhamnoside (Myricitrin) | 414666.67 | 158666.6667 | 1.31 | 2.61 |
| pme3484 | Myricetin 3-O-galactoside | 10760000.00 | 27766666.67 | 1.21 | 0.39 |
| **Catechin derivatives** | | | | | |
| pme1535 | (+)-Gallocatechin (GC) | 43400000.00 | 19950000 | 1.09 | 2.18 |
| pme1562 | Epicatechin gallate (ECG) | 33400.00 | 6949.666667 | 2.13 | 4.81 |
| **Procyanidin** | | | | | |
| pme0431 | Procyanidin A1 | 438003.00 | 1193000 | 2.02 | 0.37 |
| **Cyanidin derivatives** | | | | | |
| pmb0542 | Cyanidin 3-O-malonylhexoside | 32203.00 | 69100 | 1.83 | 0.47 |
| pme1777 | Cyanidin 3,5-O-diglucoside (Cyanin) | 22923333.33 | 7823333.333 | 1.20 | 2.93 |
| **Peonidin and its derivatives** | | | | | |
| pmb0563 | Peonidin | 49006.00 | 453003 | 1.91 | 0.11 |
| pmb2961 | Peonidin O-malonylhexoside | 1916666.67 | 684666.6667 | 1.35 | 2.80 |
| **Tricetin and its derivatives** | | | | | |
| pme3300 | Tricetin | 182800.00 | 72236.33333 | 1.89 | 2.53 |
| pma6373 | 3',4',5'-Dihydrotricetin O-hexosyl-O-hexoside | 4396666.67 | 1420333.333 | 1.43 | 3.10 |
| **Hesperetin and its derivatives** | | | | | |
| pmb0618 | 8-C-hexosyl-hesperetin O-hexoside | 173566.67 | 1584000 | 2.09 | 0.11 |
| **Syringetin and its derivatives** | | | | | |
| pmb0565 | Syringetin 3-O-hexoside | 2033333.33 | 756000 | 1.43 | 2.69 |
| **Eriodictyol and its derivatives** | | | | | |
| pmb0628 | Eriodictyol C-hexosyl-O-hexoside | 9.00 | 229672.6667 | 1.92 | 0.00 |
| **Others** | | | | | |
| pme1544 | Acacetin | 65566.67 | 28156.66667 | 1.32 | 2.33 |
| pme3250 | Biochanin A | 36830.00 | 16806 | 2.64 | 2.19 |
| pme3230 | Calycosin | 4506.00 | 10569.66667 | 1.38 | 0.43 |
| pme2984 | Isosakuranetin-7-neohesperidoside (Poncirin) | 44600.00 | 208433.3333 | 1.30 | 0.21 |
| pme0374 | Isovitexin | 168333.33 | 19569.66667 | 2.49 | 8.60 |
| pme1500 | Kumatakenin | 1028466.67 | 84793.33333 | 1.74 | 12.13 |
| pme1588 | Isorhamnetin | 43746.67 | 9446.666667 | 1.12 | 4.63 |
| pma6558 | Velutin | 1128900.00 | 103166.6667 | 1.56 | 10.94 |
