# Supplementary material for: Integrated Metabolomic and Transcriptomic Analysis of the Flavonoid Accumulation in the Leaves of Cyclocarya paliurus at Different Altitudes
Source: Front Plant Sci. 2022 Feb 8;12:794137. doi: 10.3389/fpls.2021.794137 (PMC8860981; doi:10.3389/fpls.2021.794137)
Supplement: Supplementary file 9 [file Table_6.doc]

Table S6 Overview of transcriptome sequencing of complementary DNA from *Cyclocarya paliurus* leaves. HA indicates high altitude, while LA indicates low altitude.

| Sample | Raw reads | Clean reads | Clean bases | Q20(%) | Q30(%) | GC(%) |
| --- | --- | --- | --- | --- | --- | --- |
| HA-1 | 50535242 | 45373768 | 6098531357 | 97.93% | 92.51% | 49.69% |
| HA-2 | 51370720 | 46292002 | 6260829757 | 97.94% | 92.52% | 50.75% |
| HA-3 | 18814256 | 15942348 | 2274594827 | 98.68% | 95.18% | 57.74% |
| LA-1 | 38268042 | 34908620 | 5019036992 | 99.12% | 96.59% | 57.80% |
| LA-2 | 45832324 | 43129548 | 6220550595 | 99.18% | 96.84% | 54.56% |
| LA-3 | 44614006 | 39826912 | 5348783862 | 97.80% | 92.13% | 53.59% |
| Summary | 249434590 | 225473198 | 31222327390 | 98.44% | 94.30% | 54.02% |
